# Gynecological Experiences

**Published:** 1883-04

**Authors:** 


Gynecological Experiences. A Paper Read before the California State Homœopathic Medical Society, San Francisco, November 8th, 1882. By G. M. Pease, M. D.

This pamphlet is very interesting. It shows plainly two things: (1), That Dr. Pease *believes* in Homœopathy and its therapeutics, and (2), that he keeps himself well posted as to the movements of the *enemy!*

